# Federated Learning for Thyroid Ultrasound Image Analysis to Protect Personal Information: Validation Study in a Real Health Care Environment

**DOI:** 10.2196/25869

**Published:** 2021-05-18

**Authors:** Haeyun Lee, Young Jun Chai, Hyunjin Joo, Kyungsu Lee, Jae Youn Hwang, Seok-Mo Kim, Kwangsoon Kim, Inn-Chul Nam, June Young Choi, Hyeong Won Yu, Myung-Chul Lee, Hiroo Masuoka, Akira Miyauchi, Kyu Eun Lee, Sungwan Kim, Hyoun-Joong Kong

**Affiliations:** 1 Institute of Medical & Biological Engineering Medical Research Center Seoul National University College of Medicine Seoul Republic of Korea; 2 Department of Information and Communication Engineering Daegu Gyeongbuk Institute of Science & Technology Daegu Republic of Korea; 3 Department of Surgery Seoul Metropolitan Government Seoul National University Boramae Medical Center Seoul Republic of Korea; 4 Transdisciplinary Department of Medicine and Advanced Technology Seoul National University Hospital Seoul Republic of Korea; 5 Department of Surgery Thyroid Cancer Center Gangnam Severance Hospital Seoul Republic of Korea; 6 Department of Surgery College of Medicine The Catholic University of Korea Seoul Republic of Korea; 7 Department of Otolaryngology-Head and Neck Surgery College of Medicine The Catholic University of Korea Seoul Republic of Korea; 8 Department of Surgery Seoul National University Bundang Hospital Seongnam-si, Gyeonggi-do Republic of Korea; 9 Department of Otorhinolaryngology-Head and Neck Surgery Korea Cancer Center Hospital Korea Institute of Radiological and Medical Science Seoul Republic of Korea; 10 Department of Surgery Kuma Hospital Kobe Japan; 11 Department of Surgery Seoul National University Hospital and College of Medicine Seoul Republic of Korea; 12 Department of Biomedical Engineering Seoul National University College of Medicine Seoul Republic of Korea; 13 Department of Medicine Seoul National University College of Medicine Seoul Republic of Korea

**Keywords:** deep learning, federated learning, thyroid nodules, ultrasound image

## Abstract

**Background:**

Federated learning is a decentralized approach to machine learning; it is a training strategy that overcomes medical data privacy regulations and generalizes deep learning algorithms. Federated learning mitigates many systemic privacy risks by sharing only the model and parameters for training, without the need to export existing medical data sets. In this study, we performed ultrasound image analysis using federated learning to predict whether thyroid nodules were benign or malignant.

**Objective:**

The goal of this study was to evaluate whether the performance of federated learning was comparable with that of conventional deep learning.

**Methods:**

A total of 8457 (5375 malignant, 3082 benign) ultrasound images were collected from 6 institutions and used for federated learning and conventional deep learning. Five deep learning networks (VGG19, ResNet50, ResNext50, SE-ResNet50, and SE-ResNext50) were used. Using stratified random sampling, we selected 20% (1075 malignant, 616 benign) of the total images for internal validation. For external validation, we used 100 ultrasound images (50 malignant, 50 benign) from another institution.

**Results:**

For internal validation, the area under the receiver operating characteristic (AUROC) curve for federated learning was between 78.88% and 87.56%, and the AUROC for conventional deep learning was between 82.61% and 91.57%. For external validation, the AUROC for federated learning was between 75.20% and 86.72%, and the AUROC curve for conventional deep learning was between 73.04% and 91.04%.

**Conclusions:**

We demonstrated that the performance of federated learning using decentralized data was comparable to that of conventional deep learning using pooled data. Federated learning might be potentially useful for analyzing medical images while protecting patients’ personal information.

## Introduction

Deep neural networks for image classification, object detection, and semantic segmentation have been proven to be high performance, surpassing human-level performance in some fields [1]. Deep learning for computer aided diagnosis has been frequently reported using various medical imaging modalities, such as ultrasound images, computed tomography, and magnetic resonance imaging. As in other fields, the ability for deep learning using medical images to surpass human-level performance is dependent on the volume and quality of data [2,3].

There are several challenges in the implementation of deep learning in the clinical environment. To obtain a sufficient number of medical images for high performance, medical images must be collected from multiple institutions. Personal information protection may be violated during the data collection process. Heterogeneity of data between contributing institutes is another issue that can negatively influence the performance of a deep learning network. Distribution of data varies considerably between institutions in terms of disease entities, as does the volume, location, and characteristics of medical images; this influences the performance of deep learning networks.

Federated learning is a technique used to build learning networks without the need for centralized data that is hugely advantageous in a health care context where data protection and patient confidentiality are paramount. Federated learning mitigates many systemic privacy risks by sharing with each local data source only the model and trained parameters for network training, without the need to export existing medical data sets. Network parameters that are trained with data from each local data source are aggregated in one place and are updated and sent back to each local data source. The network is trained as this process is repeatedly executed.

Although federated learning does not require the exchange of local data (ie, each medical institution’s data), it’s performance is similar to that of conventional deep learning. Federated learning has been applied to multiple open data sets such as Modified National Institute of Standards and Technology (MNIST) [4], Canadian Institute for Advanced Research (CIFAR-10) [4], and Brain Tumor Segmentation challenge (BraTS) 2018 [5,6] data sets. Various methods [4,6] have been applied to optimize the performance of federated learning. The application of federated learning for personal health information from wearable devices has also been reported [7]. These studies [4-7] demonstrated that federated learning is similar in performance to conventional deep learning (ie, data centralized training) approaches; however, they used either general image data, or if used, medical image data were few in number (for example, open medical image data sets such as BraTS 2018 contain only a few hundred images). In addition, the images were from one institution, and only one deep learning network was used. In real-world health care environments, when deep learning is applied, data distributions are frequently unbalanced.

In this study, we collected thyroid ultrasound images from medical institutions to evaluate the feasibility and performance of federated learning.

## Methods

### Thyroid Nodule Clinical Data Collection

The institutional review boards at all participating institutions (Seoul Metropolitan Government Seoul National University Boramae Medical Center, Gangnam Severance Hospital, Seoul National University Bundang Hospital, Catholic University of Korea Incheon St. Mary’s Hospital, Catholic University of Korea Seoul St. Mary’s Hospital, and Korea Cancer Center Hospital) approved this study. Representative institutional review board approval was granted by Seoul Metropolitan Government Seoul National University Boramae Medical Center (H-10-2020-195).

**Figure 1:**
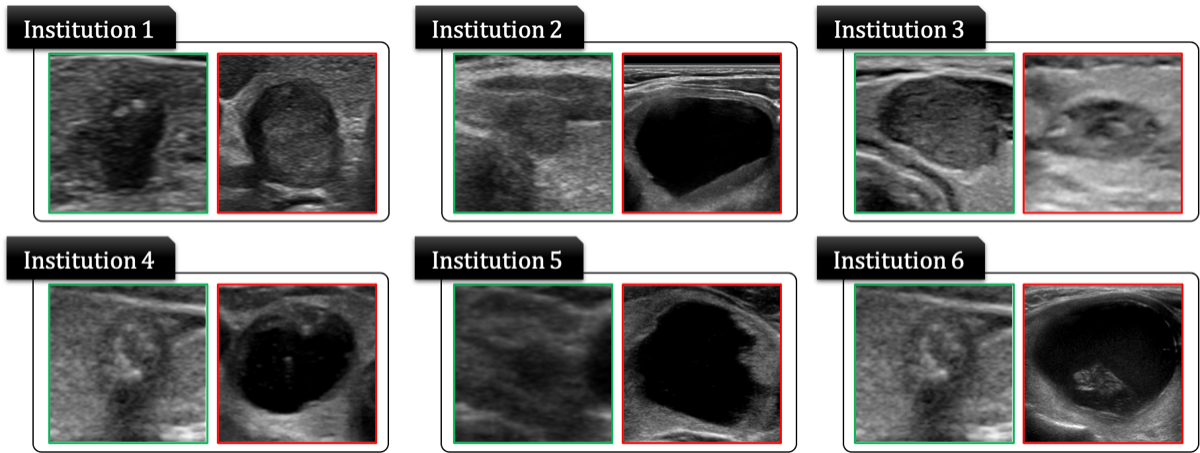
Thyroid ultrasound image data collected from 6 medical institutions to verify federated learning.

Images were collected from 6 medical institutions in captured DICOM file format (Figure 1). Of the 6 institutions, 3 used iU22 systems (Philips Healthcare), one used EPIQ 5G (Philips Healthcare), one used Prosound Alpha 7 (Hitachi Aloka), and one used Aplio 500 Platinum (Toshiba Medical Systems). Experienced surgeons at each institution labeled the images as *benign* (fine-needle aspiration cytology Bethesda Category II or benign surgical histology) or *malignant* (fine-needle aspiration cytology Bethesda Category V/VI or surgical histology of thyroid carcinoma). The images were cropped into 299×299 pixels to include typical thyroid features. The images were not augmented.

Table 1 summarizes details of the thyroid ultrasound images used in this experiment. We used 80% of each institution’s data as training data and the remaining 20% as test data. We used stratified random sampling to select the test data set. There was a total of 4300 malignant images and 2465 benign images in the total training data set and a total of 1075 malignant images and 617 benign images in the test data set. For external validation, 100 thyroid ultrasound images (50 malignant image data and 50 benign) were provided by a medical institution in Japan. We were blinded to the labeling (malignant or benign) of the images.

**Table 1:** Thyroid ultrasound image data from 6 medical institutions used to validate federated learning.

Class		Institution 1, n	Institution 2, n	Institution 3, n	Institution 4, n	Institution 5, n	Institution 6, n	Total, n
**Malignant**		**1233**	**3191**	**469**	**106**	**99**	**277**	**5375**
	Training	986	2553	375	85	79	222	4300
	Test	247	638	94	21	20	55	1075
**Benign**		**2257**	**291**	**10**	**100**	**100**	**324**	**3082**
	Training	1806	233	8	80	80	259	2466
	Test	451	58	2	20	20	65	616

In addition, to verify the performance of federated learning with external data, we collected an external test data set, which consisted of 50 malignant and 50 benign ultrasound images taken using a TUS-A500 system (Toshiba Medical System) from Kuma Hospital.

### Federated Learning System Design in a Real Health Care Environment

**Figure 2:**
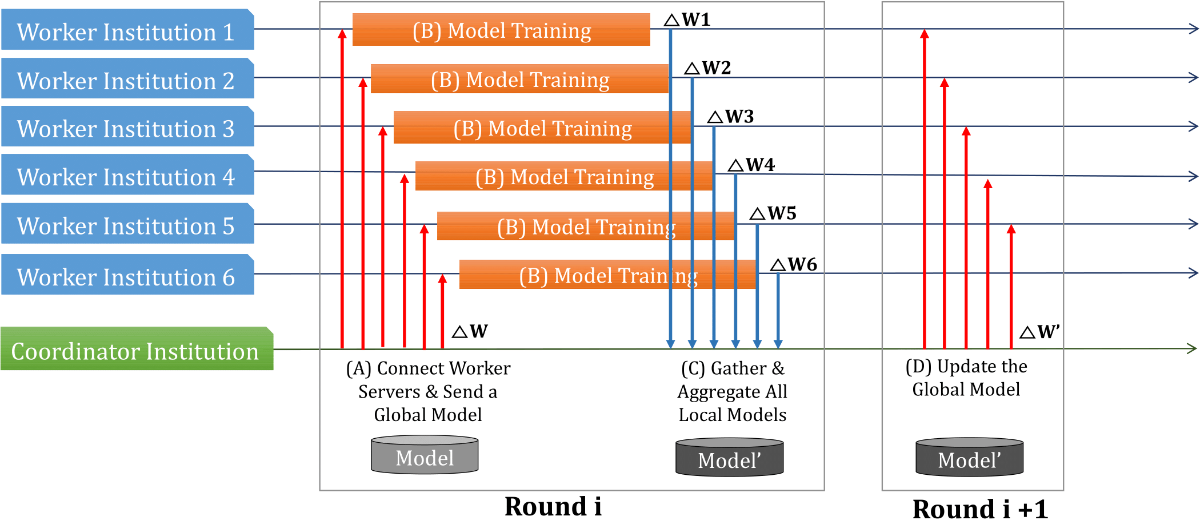
Federated learning procedure in a real-world health care environment. (A) The serverworker from each medical institution (upper 6 medical institutions) was trained with local data from their corresponding medical institution. (B) Trained parameters were sent from each institution to the coordinator. (C) The coordinator averaged the parameters received from each institution. (D) The average value was sent back to each serverworker.

We conducted federated learning experiments (Figure 2) with each institution’s serverworker (a computer system that can train deep learning algorithms with local data in the federated learning process) and the coordinator of Seoul National University Hospital to validate federated learning in a real health care environment (serverworker system at each institution: Intel 4-core 2.3 GHz i5-8259U processor, 16 GB DDR4 RAM memory, and 11 GB Nvidia RTX 2080 Ti graphics; coordinator system: 2.3 GHz i5-8259U processor, 16 GB DDR4 RAM, and 8 GB Nvidia GTX 1080). Network training was performed on the serverworkers, and then each serverworker was configured with a high-memory graphic process unit. We configured the system using the processor and external graphics processing unit for system portability. All versions of software (Python version 3.6.5; PyTorch version 1.4.0; PySyft version 0.2.5) were identical between institutions. We installed Ubuntu 18.04 LTS version on each serverworker and the coordinator system.

### Deep Learning Algorithm

We used 5 deep neural network classifiers for thyroid ultrasound image analysis: VGG19 [8], ResNet50 [9], ResNext50 [10], SE-ResNet50, and SE-ResNext50 [11]. We also used these 5 models to verify federated learning.

Stochastic optimization (ADAM) was used with the following parameters: β_1_=0.9, β_2_=0.999, ϵ=10^–8^ [12]. The initial learning rate was 0.001 which was reduced by half every 30 rounds. The mini-batch size was 32. We used a binary cross-entropy loss function to train all networks. We trained the network for 120 rounds. We used PyTorch [13] and PySyft [14] to implement and train all networks with federated learning.

### Conventional Deep Learning Using Pooled Data

After removing all patient identifying information, images from each participating institution were collected at Seoul National University Hospital to create a pooled data set. We used the pooled data set to conduct conventional deep learning. All settings were the same as those for federated learning, with the exception of those used in PySyft, and the same equipment, with the same specifications as those of the serverworker, was used. Only training data from each hospital used in the federated learning were pooled and used for conventional deep learning. The test data set was the same as that used for federated learning.

## Results

### Federated Learning Performance

**Figure 3:**
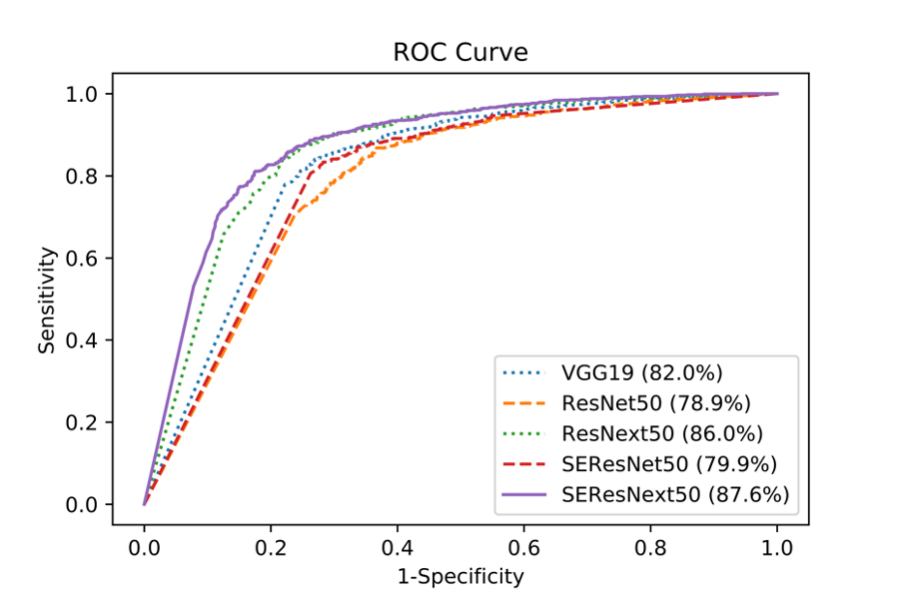
Receiver operating characteristic curves of each deep learning network for the internal test data set.

For the internal test data set, consisting of 1691 images (1075 malignant and 616 benign), and federated learning–trained deep learning algorithms, the accuracies of VGG19, SE-ResNet50, ResNet50, SE-ResNext50, and ResNext50 were 79.5%, 77.9%, 77.4%, 77.2%, and 73.9%, respectively (Table 2; Table S1 in Multimedia Appendix 1). Figure 3 shows the receiver operating characteristic curve [15] of each network for the internal test data set. Area under the receiver operating characteristic (AUROC) curve values of SE-ResNext50, ResNext50, VGG19, SE-ResNet50, and ResNet50 were 87.6%, 86.0%, 82.0%, 79.9%, and 78.9%, respectively.

**Table 2:** Thyroid classification results with federated learning with internal test data.

Deep learning algorithm	Accuracy (%)	Specificity (%)	Sensitivity (%)	PPV^a^ (%)	NPV^b^ (%)	F1 score (%)	AUROC (%)
VGG19	79.5	64.3	88.2	81.2	75.7	84.5	82.0
ResNet50	77.4	57.8	88.6	78.6	74.3	83.3	78.9
ResNext50	73.9	31.5	98.2	71.5	91.1	82.7	86.0
SE-ResNet50	77.9	56.3	90.2	78.3	76.8	83.8	79.9
SE-ResNext50	77.2	42.1	97.3	74.6	90.0	84.4	87.6

^a^PPV: positive predictive value.

^b^NPV: negative predictive value.

For the external test data set and federated learning model, the accuracies of ResNet50, SE-ResNet50, VGG19, SE-ResNext50, and ResNext50 were 76.0%, 73.0%, 69.0%, 60.0%, and 56.0%, respectively (Table 3; Table S2 in Multimedia Appendix 1). AUROC curve values of SE-ResNet50, SE-ResNext50, ResNext50, ResNet50, and VGG19 were 86.7%, 83.4%, 83.0%, 81.0%, and 75.2%, respectively.

**Table 3:** Thyroid classification results with federated learning with external test data.

Deep learning algorithm	Accuracy (%)	Specificity (%)	Sensitivity (%)	PPV^a^ (%)	NPV^b^ (%)	F1 score (%)	AUROC (%)
VGG19	69.0	52.0	86.0	64.2	78.8	73.5	75.2
ResNet50	76.0	58.0	94.0	69.1	90.6	79.7	81.0
ResNext50	56.0	12.0	100	53.2	100	69.4	83.0
SE-ResNet50	73.0	48.0	98.0	65.3	96.0	78.4	86.7
SE-ResNext50	60.0	20.0	100	55.6	100	71.4	83.4

^a^PPV: positive predictive value.

^b^NPV: negative predictive value.

### Performance of Conventional Deep Learning Using Pooled Data

For each deep learning algorithm trained with the pooled data, the accuracies of VGG19, ResNet50, ResNext50, SE-ResNet50, and SE-ResNext50 were 81.5%, 78.7%, 85.2%, 83.2%, and 85.2%, respectively (Table 4; Table S3 in Multimedia Appendix 1). The AUROC curve values of VGG19, ResNet50, ResNext50, SE-ResNet50, and SE-ResNext50 were 87.6%, 82.6%, 91.0%, 84.5%, and 91.5%, respectively.

For conventional deep learning using the pooled external test data set, the accuracies of VGG19, ResNet50, ResNext50, SE-ResNet50, and SE-ResNext50 were 71.0%, 77.0%, 80.0%, 66.0%, and 76.0%, respectively (Table 5; Table S4 in Multimedia Appendix 1). The AUROC curve values of VGG19, ResNet50, ResNext50, SE-ResNet50, and SE-ResNext50 were 79.3%, 81.2%, 89.7%, 73.4%, and 91.0%, respectively.

**Table 4:** Thyroid classification results with conventional deep learning using pooled internal test data.

Deep learning algorithm	Accuracy (%)	Specificity (%)	Sensitivity (%)	PPV^a^ (%)	NPV^b^ (%)	F1 score (%)	AUROC (%)
VGG19	81.5	62.0	92.7	81.0	83.0	86.5	87.6
ResNet50	78.7	62.8	87.7	80.5	74.6	83.9	82.6
ResNext50	85.2	72.5	92.5	85.5	84.7	88.8	91.0
SE-ResNet50	83.2	70.0	90.7	84.1	81.2	82.7	84.5
SE-ResNext50	85.3	70.9	93.5	84.9	86.2	89.0	91.5

^a^PPV: positive predictive value.

^b^NPV: negative predictive value.

**Table 5:** Thyroid classification results with conventional deep learning using pooled external test data.

Deep learning algorithm	Accuracy (%)	Specificity (%)	Sensitivity (%)	PPV^a^ (%)	NPV^b^ (%)	F1 score (%)	AUROC (%)
VGG19	71.0	56.0	86.0	66.2	80.0	74.8	79.3
ResNet50	77.0	72.0	82.0	74.5	80.0	78.1	81.2
ResNext50	80.0	72.0	88.0	75.9	85.7	81.5	89.7
SE-ResNet50	66.0	48.0	84.0	61.8	75.0	71.2	73.4
SE-ResNext50	76.0	58.0	94.0	69.1	90.6	79.7	91.0

^a^PPV: positive predictive value.

^b^NPV: negative predictive value.

## Discussion

### Principal Results

The goal of this study was to verify the performance of federated learning in a real-world health care environment. We first collected thyroid nodule data from 6 institutions and designed a federated learning system using these data. We trained each deep learning algorithm (VGG19, ResNet50, ResNext50, SE-ResNet50, and SE-ResNext50) with the federated learning system. We also trained the same deep learning algorithms using conventional deep learning techniques and compared the performance of federated learning with that of conventional deep learning.

### Comparison With Prior Work

The medical vision community is currently actively conducting diagnosis using computer-aided diagnosis [16]. To improve the performance of computer-aided diagnosis, several deep learning algorithms have been developed and applied [17-20]. Various challenges for deep learning with open data sets have been identified [21,22]. In particular, due to health care data privacy regulations, most open data sets only have a small amount of data collected from a single institution. When training and validation are carried out with only a small volume of data, the performance of a deep learning model cannot be properly evaluated, and generality cannot properly be validated. Federated learning, which can train a deep learning model without centralized data, offers a training strategy that addresses these challenges.

There have been several recent reports of the use of federated learning trained with general images [4] and medical imaging [5,6]. McMahan et al [4] published a study using federated learning with federated averaging and reported that the average parameters trained from each serverworker each round performed similarly to those of conventional deep learning and better than those of federated stochastic gradient descent; however, the study used a relatively simple model and general image data sets (MNIST and CIFAR-10). Sheller et al [5] compared federated learning, institutional incremental learning (IIL), and cyclic IIL using the BraTS 2018 data set [21]. IIL is a collaborative learning process that trains a network with data from one institution and then continues training with another institution’s data successively. One disadvantage of this model is that when the network is trained using data from another institution, the patterns trained from the previous institutions’ data are disregarded. To compensate for this shortcoming, Sheller et al [5] proposed cyclic IIL which repeats the IIL process. They used U-Net architecture [17] for brain tumor segmentation with federated learning, IIL, and cyclic IIL and demonstrated that the performance of federated learning was superior to those of IIL and cyclic IIL; however, the study applied federated learning but did not address the class imbalance or data volume imbalance problems associated with federated learning. Li et al [6] also used the BraTS 2018 data set to compare federated learning and centralized data training; they found no significant difference in performance between federated learning and centralized data training. Most federated learning studies compare federated learning with conventional deep learning only, and there are no studies using clinical data from a real-world health care environment.

The application of federated learning in our study shows that this technology has substantial potential applicability in clinical environments. First, federated learning showed performance comparable with that of conventional deep learning, despite an extremely uneven distribution of data volume from each institution. The difference between the hospital with the most data and the hospital with the least data was 17.5 fold. Moreover, the distribution of benign and malignant images was also skewed. For example, the ratio of malignant to benign images was 47:1 for institution 3, whereas it was 1:2 for institution 1. Because data distributions between hospitals are diverse, the conditions presented in this study demonstrated the applicability of federated learning in the real world and its ability to facilitate collaboration between different size institutions.

In medical image analysis, if the amount of data is insufficient, overfitting (learning from noise in data) often occurs. In such cases, only the accuracy of the internal data set is high, and deep learning algorithms cannot be rigorously evaluated. We were able to overcome the issue of overfitting by collecting images from multiple institution and by performing external validation using images from an institute in a different country. We demonstrated that federated learning is able to maximize the efficiency of medical resources and generalizability of deep learning algorithms using data from different size medical institutions (with various imaging devices and different patient groups). This represents scenarios in real-world health care environments [23-26].

In our study, federated learning training took at least 4 times longer than that of conventional deep learning. The training time for federated learning varied depending on the peripheral environment such as internet speed and temperature of graphics process unit. The performance of federated learning may be enhanced with more images or data augmentation. The ideal volume of data and the distribution of data contributed by each institution for peak performance of federated learning is also not yet known. Further investigation into the optimal training environment, training time, data volume, data distribution, and state-of-the-art deep learning algorithms is required for federated learning.

As shown in Table 5, we noted that when thyroid nodules were classified by a conventional deep learning model, the number of malignant calls was extremely high. The same trend is frequently observed in the literature [20,27-29]. As shown in Table 3, we also found this trend to be prominent in federated learning. Because deep learning is a black box [30], we were unable to determine the potential reasons for this tendency, but we plan to investigate this phenomenon in the future.

### Limitations

This study has several limitations. First, we presented the results of federated learning used in a specific context in terms of the number of participating institutions, and the number and ratio of benign and malignant images. Thus, the generalizability of the results in terms of the performance of federated learning is not known and warrants further investigation. We also used thyroid ultrasound images, which are relatively easy to analyze compared to those from computed tomography, magnetic resonance imaging, and histopathology sections. Results may not be generalizable across different imaging modalities. In future work, comparisons of federated learning with unequal data distribution, data augmentation, one-shot learning are required to explore the implications of data imbalance.

### Conclusions

We demonstrated that the performance of federated learning using a shared training model and parameters from 6 institutions was comparable with that of conventional deep learning using pooled data. Federated learning is highly generalizable because it can effectively utilize data collected from different environments despite data heterogeneity. Federated learning has the potential to mitigate many systemic privacy risks by sharing only the model and parameters for training without the need to export existing medical data sets.

## Appendix

Multimedia Appendix 1 Tables with additional information.

## Abbreviations

AUROC: area under the receiver operating characteristic curve
BraTS: Brain Tumor Segmentation challenge data set
CIFAR: Canadian Institute for Advanced Research
IIL: institutional incremental learning
MNIST: Modified National Institute of Standards and Technology
