# Supplementary material for: Federated Learning for Thyroid Ultrasound Image Analysis to Protect Personal Information: Validation Study in a Real Health Care Environment
Source: JMIR Med Inform. 2021 May 18;9(5):e25869. doi: 10.2196/25869 (PMC8170555; doi:10.2196/25869)
Supplement: Multimedia Appendix 1 [file medinform_v9i5e25869_app1.docx]

**Multimedia Appendix 1. Tables with additional information.**

**Table S1.** Thyroid classification results with federated learning with internal test data. The best performance is in **bold**.

| Deep Learning Algorithm | VGG19 | ResNet50 | ResNext50 | SE-ResNet50 | SE-ResNext50 |
| --- | --- | --- | --- | --- | --- |
| Accuracy | **79.5%** (1344/1691) | 77.4% (1308/1691) | 73.9% (1250/1691) | 77.9% (1317/1691) | 77.2% (1305/1691) |
| Specificity | **64.3%** (396/616) | 57.8% (356/616) | 31.5% (194/616) | 56.3% (347/616) | 42.1% (259/616) |
| Sensitivity | 88.2% (948/1075) | 88.6% (952/1075) | **98.2%** (1056/1075) | 90.2% (970/1075) | 97.3% (1046/1075) |
| PPV | **81.2%** (948/1168) | 78.6% (952/1212) | 71.5% (1056/1478) | 78.3% (970/1239) | 74.6% (1046/1403) |
| NPV | 75.7% (396/523) | 74.3% (356/479) | **91.1%** (194/213) | 76.8% (347/452) | 90.0% (259/288) |
| F1 score | **84.5%** | 83.3% | 82.7% | 83.8% | 84.4% |
| AUROC | 82.0% | 78.9% | 86.0% | 79.9% | **87.6%** |

**Table S2.** Thyroid classification results with federated learning with external test data. The best performance is in **bold**.

| Deep Learning Algorithm | VGG19 | ResNet50 | ResNext50 | SE-ResNet50 | SE-ResNext50 |
| --- | --- | --- | --- | --- | --- |
| Accuracy | 69.0% (69/100) | **76.0%** (76/100) | 56.0% (56/100) | 73.0% (73/100) | 60.0% (60/100) |
| Specificity | 52.0%  (26/50) | **58.0%**  (29/50) | 12.0%  (6/50) | 48.0% (24/50) | 20.0%  (10/50) |
| Sensitivity | 86.0%  (43/50) | 94.0%  (47/50) | **100.0%** (50/50) | 98.0% (49/50) | 100.0% (50/50) |
| PPV | 64.2%  (43/67) | **69.1%**  (47/68) | 53.2%  (50/94) | 65.3% (49/75) | 55.6%  (50/90) |
| NPV | 78.8%  (26/33) | 90.6%  (29/32) | **100.0%**  (6/6) | 96.0% (24/25) | 100.0% (10/10) |
| F1 score | 73.5% | **79.7%** | 69.4% | 78.4% | 71.4% |
| AUROC | 75.2% | 81.0% | 83.0% | **86.7%** | 83.4% |

**Table S3.** Thyroid classification results with conventional deep learning using pooled internal test data. The best performance is in **bold**.

| Deep Learning Algorithm | VGG19 | ResNet50 | ResNext50 | SE-ResNet50 | SE-ResNext50 |
| --- | --- | --- | --- | --- | --- |
| Accuracy | 81.5% (1379/1691) | 78.7% (1360/1691) | 85.2% (1441/1691) | 83.2% (1406/1691) | **85.3%** (1442/1691) |
| Specificity | 62.0% (382/616) | 62.8% (387/616) | **72.5%** (447/616) | 70.0% (431/616) | 70.9% (437/616) |
| Sensitivity | 92.7% (997/1075) | 87.7% (943/1075) | 92.5% (994/1075) | 90.7% (975/1075) | **93.5%** (1005/1075) |
| PPV | 81.0% (997/1231) | 80.5% (943/1172) | **85.5%** (994/1163) | 84.1% (975/1160) | 84.9% (1005/1163) |
| NPV | 83.0% (382/460) | 74.6% (387/519) | 84.7% (447/528) | 81.2% (431/531) | **86.2%** (437/528) |
| F1 score | 86.5% | 83.9% | 88.8% | 82.7% | **89.0%** |
| AUROC | 87.6% | 82.6% | 91.0% | 84.5% | **91.5%** |

**Table S4.** Thyroid classification results with conventional deep learning using pooled external test data. The best performance is in **bold**.

| Deep Learning Algorithm | VGG19 | ResNet50 | ResNext50 | SE-ResNet50 | SE-ResNext50 |
| --- | --- | --- | --- | --- | --- |
| Accuracy | 71.0% (69/100) | 77.0% (77/100) | **80.0%** (80/100) | 66.0% (66/100) | 76.0% (76/100) |
| Specificity | 56.0%  (28/50) | **72.0%**  (36/50) | **72.0%**  (36/50) | 48.0% (24/50) | 58.0%  (29/50) |
| Sensitivity | 86.0%  (43/50) | 82.0%  (41/50) | 88.0%  (44/50) | 84.0% (42/50) | **94.0%**  (47/50) |
| PPV | 66.2%  (43/65) | 74.5%  (41/55) | **75.9%**  (44/58) | 61.8% (42/68) | 69.1%  (47/68) |
| NPV | 80.0%  (28/35) | 80.0%  (36/45) | 85.7%  (36/42) | 75.0% (24/32) | **90.6%**  (29/32) |
| F1 score | 74.8% | 78.1% | 81.5% | 71.2% | **79.7%** |
| AUROC | 79.3% | 81.2% | 89.7% | 73.4% | **91.0%** |
